# Optimal experimental designs for dose–response studies with continuous endpoints

**DOI:** 10.1007/s00204-014-1335-2

**Published:** 2014-08-26

**Authors:** Tim Holland-Letz, Annette Kopp-Schneider

**Affiliations:** Biostatistics Division, German Cancer Research Center (DKFZ), Im Neuenheimer Feld 280, 69120 Heidelberg, Germany

**Keywords:** D-optimal design, Dose response modelling, Log-logistic function, Log-normal function, Weibull function, 3T3/NHK guideline

## Abstract

In most areas of clinical and preclinical research, the required sample size determines the costs and effort for any project, and thus, optimizing sample size is of primary importance. An experimental design of dose–response studies is determined by the number and choice of dose levels as well as the allocation of sample size to each level. The experimental design of toxicological studies tends to be motivated by convention. Statistical optimal design theory, however, allows the setting of experimental conditions (dose levels, measurement times, etc.) in a way which minimizes the number of required measurements and subjects to obtain the desired precision of the results. While the general theory is well established, the mathematical complexity of the problem so far prevents widespread use of these techniques in practical studies. The paper explains the concepts of statistical optimal design theory with a minimum of mathematical terminology and uses these concepts to generate concrete usable D-optimal experimental designs for dose–response studies on the basis of three common dose–response functions in toxicology: log-logistic, log-normal and Weibull functions with four parameters each. The resulting designs usually require control plus only three dose levels and are quite intuitively plausible. The optimal designs are compared to traditional designs such as the typical setup of cytotoxicity studies for 96-well plates. As the optimal design depends on prior estimates of the dose–response function parameters, it is shown what loss of efficiency occurs if the parameters for design determination are misspecified, and how Bayes optimal designs can improve the situation.

## Introduction

The investigation of a dose–response relationship requires measurement of responses at different dose levels. A frequently occurring question in this context is how to optimally allocate measurements to selected dose levels, i.e., the choice of the experimental study design. The size and the design of a toxicological study largely determine the costs of the investigation. Other restrictions may be imposed by physical or administrative constraints, such as the total number of measurements in the study, e.g., using a 96-well plate.

Statistical optimal design theory in general covers the choice of experimental settings in order to estimate parameters of interest as precisely as possible, thus minimizing the number of experimental units needed. The theory is well developed; however, it uses quite sophisticated mathematical methods not easily accessible without a mathematical background. Thus, optimal design theory is very rarely used in practical studies, even though there are several areas in preclinical and clinical development where the theoretical results fit the practical situation very well. Furthermore, while the theoretical concepts are sometimes complex, the practical results are often quite usable and intuitively plausible. Prime candidates for statistical optimization are studies assessing dose–response, toxicological and pharmacokinetic properties of chemicals. In this paper, the focus will be on toxicological studies.

In their investigation about toxicity study designs, Slob et al. (2005) use a computer simulation approach to investigate the performance of the typical dose–response study design for determination of the no-observed-adverse-effect-level (NOAEL), when the study aim is to estimate a benchmark dose (BMD) in continuous endpoints. The typical design for a NOAEL study involves one control and three dose groups with five animals each. Slob et al. argue that in order to minimize the risk of inadequate dose placement, a larger number of dose groups should be used, even though the number of animals in each dose group will decrease. BMD estimation is based on a model fit to the data, rather than statistical tests with pairwise comparison of dose to control, which is the strategy for NOAEL estimation. As noted by Rhomberg (2005), the optimal study design crucially depends on the purpose of the toxicological study. In order to retain maximum flexibility, the present investigation will concentrate on those designs, which will allow the most effective model fit and thus result in precise estimates of most characteristic toxicological values. In design theory, such designs are called D-optimal.

In the present paper, we actually propose optimal designs for dose–response studies in toxicology with continuous endpoints on the basis of commonly used nonlinear functions for dose–response modeling as described in Ritz (2010), for application, see, e.g., Clothier et al. (2013). The aim of our paper is to both explain the general ideas behind some important parts of optimal design theory as well as actually construct usable optimal designs for several dose–response functions commonly applied in toxicology. Furthermore, we will compare these designs to the design principles recommended by relevant guidelines. It is not the aim to actually show or derive the mathematical details and theory; other resources are available for this [see, e.g., Fedorov (1972), Pazman (1986), Li and Majumdar (2008), Yu (2010) for the algorithm, and Fedorov and Leonov (2001) for some applications closer to toxicology].

Dose–response relationships in toxicology are usually described by nonlinear models. When statistical optimal designs are derived for nonlinear models, the design can only be derived with assumptions for the model parameters. Hence, these designs are referred to as “locally optimal designs.” The dependence on assumptions for model parameters has motivated research to suggest pragmatic solutions, which are less susceptible to parameter misspecification. This so-called Bayesian design approach will also be addressed in this manuscript.

The present paper is organized as follows: The modeling functions are introduced in “Dose–response functions in toxicology” section. The required parts of optimal design theory are explained in “Optimal experimental design” section, and designs for several dose–response functions are provided in “Optimal designs for specific situations” section. The Bayesian approach is described in “Bayes optimal designs” section, while a comparison of the optimal designs to the OECD guideline for cytotoxicity testing is presented in “Comparison to the 3T3/NHK cytotoxicity testing guideline” section.

## Dose–response functions in toxicology

Parameter estimation from dose–response experiments is usually based on fitting a nonlinear model to the dose–response data using the maximum likelihood or (nonlinear) least-squares approach. The general nonlinear regression model is given by

$$y = f(x) + {\text{random}}\;{\text{error}}$$

where random errors are assumed to be independently normally distributed with mean zero and standard deviation *σ*. The dose is denoted by *x* and the response by *y*. Hence, *f*(*x*) describes the mean response at concentration *x* (>0). Different functions can be used to model the concentration–response relationship. In this paper, we will consider three common types of dose–response relationships as described in Ritz (2010), in particular the log-logistic, the log-normal and the Weibull relationship.

Log-logistic:

1 $$f(x;b,c,d,e) = c + \frac{d - c}{1 + \exp (b(\log (x) - \log (e)))}$$

Log-normal:

2 $$f(x;b,c,d,e) = c + (d - c)\varPhi ( - b(\log (x) - \log (e)))$$

Weibull:

3 $$f(x;b,c,d,e) = c + (d - c)\exp ( - \exp (b(\log (x) - \log (e))))$$

Here, *exp* denotes the exponential function to base Euler’s constant ≈2.718, *log* the natural logarithm and $\varPhi$ the cumulative distribution function of the standard normal distribution. The parameters *c* and *d* correspond to the lower and upper limits for mean response, respectively, and they are in the same units as the endpoint itself. For the log-logistic and the log-normal model, the parameter *e* corresponds to the ED50, the dose leading to halfmaximal response, whereas for the Weibull model, *e* corresponds to the inflection point of the curve. The parameter *b* determines the slope of the dose–response curve at dose *e*. The model parameters are typically unknown, and they are obtained by nonlinear least-squares fit to experimental data. Note that Ritz (2010) differentiates two variants of the Weibull function, which have different biological interpretations, but are identical in the mathematical sense. Our results apply to both variants. Furthermore, for the log-normal model, we chose a variant with −*b* in order to obtain a decreasing function for a positive *b*.

For all functions, we call the parameterization *c* = 0, *d* = 1, *b* = 1 and *e* = 1 the standard parameterization. In this situation, the mean response values are contained between 0 and 1, and the ED50 is at the dose of 1, i.e., log ED50 is 0.

**Fig. 1:**
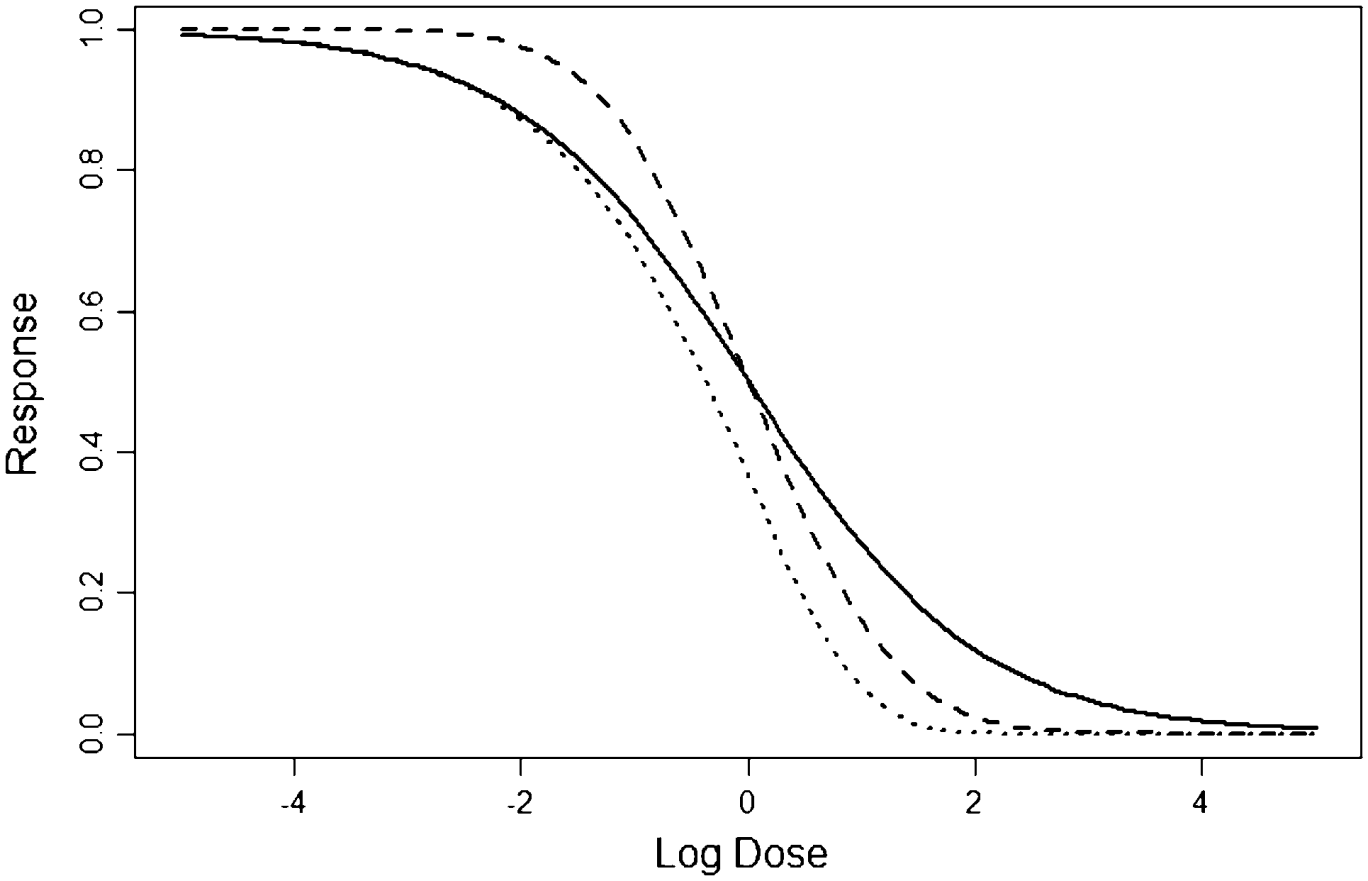
Log-logistic (*solid line*), log-normal (*dashed line*) and Weibull (*dotted line*) dose–response curve for standard parameter values

Figure 1 shows the three functions under standard parameterization and illustrates that the three functions are similar but not identical. The depicted functions are decreasing. However, increasing functions result from either switching the roles of *c* and *d*, or by choosing a negative *b*, i.e., *b* = −1.

## Optimal experimental design

In a specific study, a specified total number of observations can be taken at any or all available dose levels, and the four parameters *b*, *c*, *d* and *e* are to be estimated. The purpose of optimal experimental design is to select the dose levels to be used for each observation in order to estimate the four parameters with optimal precision. A specific selection of dose levels for all *n* observations is called a *design*.

In the following, we shortly summarize results from optimal design theory. Since derivation of some of the results used here requires somewhat complex mathematics, we refer to the books by Fedorov (1972) and Pazman (1986) for the mathematical details.

### Estimation errors

In order to identify optimal dose levels, one has to consider the effect of the dose levels on the precision of the parameter estimates, which in the mathematical sense is reflected by the variance of the estimators. For nonlinear models, this variance is constructed using a matrix *F* containing the derivatives of the dose–response function *f* in the direction of all the parameters of interest, with one column for each of the *n* measurements at doses *x*_1_,…, *x*_*n*_:

$$F = \left( {\begin{array}{*{20}l} {\frac{{\partial f(x_{1} )}}{\partial b}} \hfill & \cdots \hfill & {\frac{{\partial f(x_{n} )}}{\partial b}} \hfill \\ {\frac{{\partial f(x_{1} )}}{\partial c}} \hfill & \cdots \hfill & {\frac{{\partial f(x_{n} )}}{\partial c}} \hfill \\ {\frac{{\partial f(x_{1} )}}{\partial d}} \hfill & \cdots \hfill & {\frac{{\partial f(x_{n} )}}{\partial d}} \hfill \\ {\frac{{\partial f(x_{1} )}}{\partial e}} \hfill & \cdots \hfill & {\frac{{\partial f(x_{n} )}}{\partial e}} \hfill \\ \end{array} } \right)$$

It can then be shown (see, e.g., Searle 1971 chapter 3.3) that for both *least squares* and *maximum likelihood* estimation, the variance V for a parameter estimate of the column vector (*b*, *c*, *d*, *e*) is approximately given by

$$V \approx \sigma^{2} \left( {FF^{\text{T}} } \right)^{ - 1} ,$$

where *F* ^T^ indicates the transposed (mirrored) version of *F*. *F* and *F* ^T^ are multiplied via matrix multiplication and ()^−1^ indicates the matrix inverse. As noted in “Dose–response functions in toxicology” section, *σ* denotes the standard deviation of the errors of any observation.

As *F* is constructed from derivatives, its elements will get larger the more the response is changed when the parameters change. Correspondingly, as *V* includes a matrix inverse, the variance of the parameter estimates will decrease in this situation. Therefore, as a general principle, dose levels should be selected as those doses where variations in the parameters result in the largest possible changes of the resulting response.

The central part of the variance is *FF* ^T^, and it is called the *information matrix M*. It is the only part depending on the dose levels *x*_1_,…, *x*_*n*_ and maximizing it will thus guarantee minimal variance and allow optimal parameter estimation.

### D-optimality

The 4 × 4-matrix *M* = *FF* ^T^ describes the information of a given design (set of *n* dose levels) for the four parameters of interest, and it is the aim of optimal design theory to maximize this matrix. Unfortunately, it is not possible to choose a design which simultaneously maximizes the information for all parameters; designs that are optimal for one parameter are generally suboptimal for other parameters. The most common solution to this problem is to select a design which maximizes a function of the matrix which yields some kind of average information for all parameters. This is achieved by the *determinant* of the information matrix *M* = *FF* ^T^, which roughly corresponds to the geometric mean of the variances of the four parameter estimates. This approach is called *D*-*optimality*. Other variants like *c*-*optimality* (optimize estimation of a linear combination of parameters) or *a*-*optimality* (optimize the sum of individual variances of parameter estimates) exist, but these are less common and will not be covered here. Such functions which reduce the information matrix to a single value are called *information functions*.

Unfortunately, the resulting maximization problems usually cannot be solved analytically. However, algorithmic approaches exist, and it is possible to formulate a condition which, for any given design, can confirm whether this design is in fact optimal or not. The latter is called an *equivalence theorem* (see Kiefer 1974).

### Equivalence theory

To explain the general idea behind equivalence theory, we first have to introduce the concept of an elementary design, which is a (hypothetical) design with all measurements at the same dose level. If *m* different dose levels are possible, there are *m* elementary designs. While an elementary design does not allow estimation of four parameters, it is still possible to calculate an information matrix for each elementary design. We can then check whether each elementary information matrix contains information not included adequately in the given design, and thus, whether the corresponding dose level should be given more weight in the existing design. Formally, this is done using matrix-wise derivatives, which is a somewhat complex mathematical concept measuring by how much a function of a matrix will change when the matrix is changed.

In a nutshell, the idea behind the equivalence theorem is the following: First, take the information function of any given design and calculate the matrix-wise derivative of this function in direction of the information matrix of every elemental design (separately). This derivative will be positive only if the dose level corresponding to the elementary design would improve the performance of the current design. Thus, if none of the derivatives are larger than or equal to zero, the proposed design is already optimal.

Actually calculating the derivatives requires advanced matrix algebra (see Pazman 1986). Fortunately, it results in an easily testable condition. We write *A*_1_,…, *A*_*m*_ for the information matrices of the elementary designs for the *m* possible dose levels *x*_1_,…, *x*_*m*_ and can then express the condition to be tested (the equivalence theorem) as follows:

A design with information matrix *M* is D-optimal for the estimation of all four parameters, if and only if

4 $${\text{tr}}(A_{j} M^{ - 1} )/4 \le 1\quad {\text{for}}\;{\text{all}}\;j\;{\text{from}}\;1\;{\text{to}}\;m.$$

The notation *tr* indicates the trace of a matrix and is defined simply as the sum of its diagonal elements. Condition (4) can be checked for any candidate design to determine whether it is optimal or not. We will use an algorithmic approach and condition (4) to find and confirm optimal designs for the dose–response functions described in “Dose–response functions in toxicology” section.

### Algorithm for selection of optimal design

We will use a stepwise algorithm first suggested by Titterington (1976). The convergence of this multiplicative algorithm toward the optimal design has been proven quite generally by Yu (2010).

One property of the equivalence theorem is the following: If more observations on a dose level *x*_*j*_ would improve the performance of the design under consideration, then the left side of Eq. (4) will actually be larger than one for this dose level. Correspondingly, dose levels which should get less consideration will have values less than one. Thus, we can determine for any design which dose levels to add and which to remove.

The idea of the algorithm is to start with a simple design, usually an equal weights design. The weights of all the dose levels in the current design are then multiplied by the value obtained for the left side in Eq. (4). More formally, if we write the weights for each dose level in our current design at step *i* as (*w*_1_^*i*^,…,*w*_*m*_^*i*^), then weights for the next iteration of the design will be given by

5 $$w_{j}^{i + 1} = w_{j}^{i} * {\text{tr}}(A_{j} M^{{i^{ - 1} }} )/4,\quad {\text{for}}\;{\text{all}}\;{\text{dose}}\;{\text{levels}}\;j = 1, \ldots ,m.$$

The new designs will always be better than the previous ones and will converge toward the optimal one (see Yu 2010).

The optimal design is therefore a set of dose levels and weights. For the design of an actual experiment, the number of replicates per dose level is determined from the weights of the optimal design, using standard rounding procedures. Usually, the loss of precision due to rounding is minor (see Pukelsheim and Rieder 1992).

### Efficiencies of designs

In the following, we will compare designs regarding their performance. The aim of optimal designs is the estimation of model parameters with optimal precision, i.e., the minimization of an information function. Thus, the appropriate way to measure the efficiency of a specific design is to put its information function in relation to the information function of the optimal design. The efficiency of a D-optimal design is defined as

$${\text{Efficiency}}\left( {\text{Design}} \right) = \sqrt[k]{{\frac{{{\text{Determinant}}\left( {M_{\text{Design}} } \right)}}{{{\text{Determinant}}\left( {M_{\text{OptimalDesign}} } \right)}}}}$$

where *k* denotes the number parameters in the model, which is four in our case. Hence, the optimal design itself has an efficiency of 100 %. An efficiency of 50 % means that twice the number of observations are needed to obtain the same precision. In the following, we will always consider the situation of a standard design of 100 observations and compare the required sample sizes of competing designs against this. Of course, results can be rescaled to any desired sample size.

## Optimal designs for specific situations

We will now apply the algorithm to the three dose–response functions discussed in “Dose–response functions in toxicology” section. In all cases, the derivatives of the dose–response function with respect to its four parameters are needed to determine the matrices *F*, *A*_*j*_ and *M*.

### Log-logistic function

In a first step, the vector of derivatives of the log-logistic function

$$f(x;b,c,d,e) = c + \frac{d - c}{1 + \exp (b(\log (x) - \log (e)))}$$

is needed. For ease of notation, we will subsequently write *E* for the term $\exp (b(\log (x) - \log (e)))$, i.e., the log-logistic function is abbreviated as

$$f(x;b,c,d,e) = c + \frac{d - c}{1 + E}.$$

The derivatives are then given by:

$$\frac{\partial f(x)}{\partial c} = 1 - \frac{1}{1 + E};\quad \frac{\partial f(x)}{\partial d} = \frac{1}{1 + E};$$

$$\frac{\partial f(x)}{\partial b} = - \frac{d - c}{{(1 + E)^{2} }} * (\log (x) - \log (e)) * E;\quad \frac{\partial f(x)}{\partial e} = \frac{d - c}{{(1 + E)^{2} }} * \frac{b}{e} * E.$$

We can now apply the multiplicative algorithm.

**Fig. 2:**
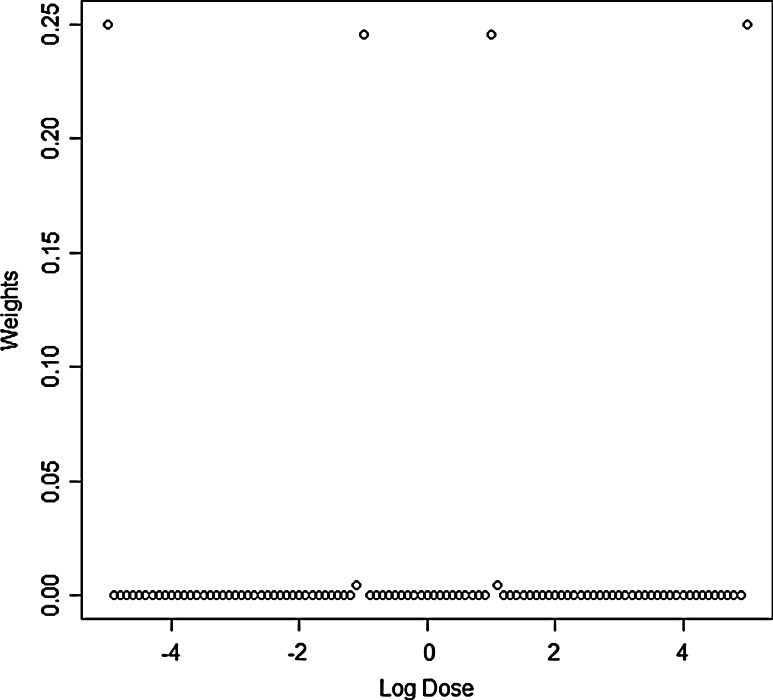
Weights for different dose levels in the log-logistic model under standard parameterization

We start with the function in standard parameterization, i.e., *b* = 1, *c* = 0, *d* = 1 and *e* = 1. In this parameterization, the dose range of interest is covered by log dose levels between −5 and 5 (cf. Fig. 1). We divide the dose range into 101 steps of width 0.1 on a logarithmic scale: log values of −5.0, −4.9,…, −0.1, 0, 0.1,…, 4.9, 5.0. We call this dose range the *standard set of dose levels*. Running the algorithm for 1,000 iterations results in weights shown in Fig. 2.

All weights are 0 with exception of six dose levels, two of which have a very small weight. Neglecting these two dose levels has negligible effect on the performance of the design, and we thus propose to select the four major dose levels only. These are −5, −1, 1 and 5 on the log dose scale (see also Table 1), corresponding to original dose levels of 0.007, 0.37, 2.72 and 148.4 units. Each dose level is given the same weight 0.25 and should thus be used in a quarter of the available replicates in the experiment. Comparing the selected dose levels to the dose–response curve in Fig. 1, we conclude that measurements should be taken at the largest and smallest available dose level, as well as at the beginning and the end of the nearly linear descent in the center of the curve. The optimality of this design has been confirmed with the equivalence theorem. Note that the need for the smallest and largest dose level as well as the four point nature of the design can actually be proven mathematically for the log-logistic function (see Li and Majumdar 2008).

**Table 1:** Optimal designs for standard parameterization

Model	Log dose 1	Log dose 2	Log dose 3	Log dose 4
Log-logistic	−5	−1	1	5
Log-normal	−5	−0.7	0.7	5
Weibull	−5	−1	0.5	5

Dose levels for all three models and the standard parameterization *c* = 0, *d* = 1, *b* = 1 and *e* = 1. All dose levels are to be used for 25 % of replicates

Dose levels are independent of *c* and *d*

Transformation of log doses for general “*b*” or “*e*”: $\log \left( {x_{new} } \right) = \log \left( e \right) + \frac{1}{b}\log \left( {x_{\text{standard}} } \right)$

As a theoretical consideration, this optimal design could be compared to an artificial design with equal weights for all 101 dose levels, i.e., a design with equal coverage of the complete dose range. Efficiency of such a design would be 78.5 %, that is, if the optimal design had 100 observations, the equally spaced design would require 100/0.785 = 128 observations to get the same level of precision.

Recall that the dose levels and weights of the optimal design were derived for the standard parameterization. However, for the general situation with parameters *b*, *c*, *d* and *e*, this design can be adapted easily by transformation of the dose range and the doses to be used by

6 $$x_{\text{new}} = e \cdot x_{\text{standard}}^{\frac{1}{b}}$$

where *x*_standard_ are the nonlogarithmic dose levels, which correspond to the standard set of dose levels (101 log doses equally spaced between −5 and 5). The reasoning is the following: Since the parameters *c* and *d* appear in the determinant only as a multiplicative factor, they are irrelevant for the optimal design. Furthermore, the parameters *b* and *e* only appear in the determinant either as a multiplicative factor or in the term $b\left( {\log (x) - \log (e)} \right) = b \cdot \log \left( \frac{x}{e} \right)$ which we call *L*. However, replacing all dose levels *x*_standard_ with new dose levels $x_{\text{new}} = e \cdot x_{\text{standard}}^{\frac{1}{b}}$ will result in $L = b \cdot \log \left( {\frac{{x_{\text{new}} }}{e}} \right) = 1 \cdot \log \left( {\frac{{x_{\text{standard}} }}{1}} \right)$, which are the exact same values of the term *L* as under standard parameterization. Hence, the same information matrices will result for all dose levels.

Thus, a design using the optimal dose levels from the standard parameterization transformed in this way will be D-optimal for a design space of similarly transformed dose levels. Furthermore, this new design space is a sensible one as it will again cover the important part of the dose–response curve in exactly the same way as the standard set of dose levels.

All transformations refer to the original dose levels, not the log doses. For convenience, the transformation for doses on the log scale is given by $\log \left( {x_{\text{new}} } \right) = \log (e) + \frac{1}{b}\log \left( {x_{\text{standard}} } \right).$

### Log-normal function

Exactly the same considerations as before can also be applied to the log-normal function

$$f(x;b,c,d,e) = c + (d - c)\varPhi ( - b(\log (x) - \log (e))).$$

In this case, we write *g*(*x*) for the density function of the standard normal distribution and as before *L* for $b(\log (x) - \log (e))$ (note that exp(*L*) = *E*). The derivatives are then given by

$$\frac{\partial f(x)}{\partial c} = 1 - \varPhi ( - L)\quad \frac{\partial f(x)}{\partial d} = \varPhi ( - L)$$

$$\frac{\partial f(x)}{\partial b} = - (d - c) * \frac{L}{b} * g( - L)\quad \frac{\partial f(x)}{\partial e} = (d - c) * \frac{ - b}{e}*g( - L).$$

Optimal designs under standard parameterization were derived in the same way as for the log-logistic model. Again, a four point design was obtained, with a structure very similar to the log-logistic model and the four log dose levels of −5.0, −0.7, 0.7 and 5.0, each to be used on 25 % of the replicates (see Table 1). For comparison, for standard parameterization, the efficiency of the equal weights design in this model is 83.3 % compared with the optimal design (120 required observations).

As in “Log-logistic function” section, the four parameters only appear either as multiplicative constants or in the term $L = b(\log (x) - \log (e))$. Deviations in the parameters from the standard parameterization can thus be compensated in the exact same way by replacing all dose levels through formula (6).

### Weibull function

As a third situation, we will consider the Weibull function

$$f(x;b,c,b,e) = c + \left( {d - c} \right)\exp \left( { - \exp \left( {b\left( {\log \left( x \right) - \log \left( e \right)} \right)} \right)} \right).$$

Writing as before $L = b(\log (x) - \log (e))$ and $E = \exp (b(\log (x) - \log (e)))$, the derivatives are then given by

$$\frac{\partial f(x)}{\partial c} = 1 - \exp ( - E);\quad \frac{\partial f(x)}{\partial d} = \exp ( - E);$$

$$\frac{\partial f(x)}{\partial b} = - (d - c) * \exp ( - E) * E*\frac{L}{b};\quad \frac{\partial f(x)}{\partial e} = (d - c) * \exp ( - E) * E * \frac{b}{e}.$$

The resulting optimal design points are log dose levels of −5.0, −1.0, 0.5 and 5.0, similar to the other models, but with partly asymmetrical dose levels (see Table 1). Efficiency of the equal weights design under standard parameterization is 80.3 % compared with the optimal design (i.e., 125 observations instead of 100).

As before, deviations in the parameters from standard parameterization can be compensated replacing all dose levels through formula (6).

### Performance with misspecified parameters

All optimal designs given in the previous sections assume prior knowledge about the parameters to be expected and are optimal only when these parameters are indeed the true parameters (so-called *locally optimal* designs, Chernoff 1953). Consequently, they will be less efficient when these prior guesses substantially deviate from the truth. However, when the deviations are only moderate, this loss of efficiency of the designs might be minor and the designs might still allow reasonably precise parameter estimation. As the designs do not depend on *c* and *d*, we only investigated deviations for *b* and *e*. In all cases, the standard parameterization was used to derive a design, and the efficiency of this design was calculated under different true values of the parameters *b* and *e*. Recall that an efficiency of a given design of *x* % means that only *x* % of the number of subjects would be needed for the same precision if the optimal design was used instead of the given design.

Results for the number of required observations compared to an optimal setup with 100 observations are shown for several variations of the true parameters *b* and *e* in Table 2. Note that the parameters *c* and *d* do not affect the design.

**Table 2:** Required sample sizes of the optimal design for the standard parameterization when the true parameters are not standard, compared with the optimal design with 100 observations under these parameters

True *b*	True *e*	Required *n* log-logistic	Required *n* log-normal	Required *n* Weibull
1	1	100 (reference)	100 (reference)	100 (reference)
0.9	1	101	101	101
1	0.9	100	101	100
0.9	0.9	101	101	101
1.1	1.1	100	101	101
0.5	1	110	116	114
1	0.5	109	127	134
0.5	0.5	112	122	111
1	2	109	127	122
2	1	132	147	138
2	2	164	385	189

We conclude that the proposed designs are still efficient when there are minor misspecifications of the parameters. However, in case of large deviations, especially an increase in the parameter *b*, a substantial loss in efficiency is observed. Reason for this is that a very steep slope of the dose–response curve will cause the relevant part of the curve to entirely fall into the dose range between the two middle doses. In this case, *b* can no longer be reliably estimated. A design with many equally spaced dose levels might be less affected by this phenomenon. In fact, in case of the log-normal model and true *b* as well as true *e* both equal to 2, a hypothetical design with equal weights on all 101 dose levels (see “Log-logistic function” section) requires only 148 observations, while using the optimal design for the wrongly assumed standard model 385 observations would be required. Again, these numbers are both in comparison with an optimal design with 100 observations. Thus, while the optimal design is superior to an equally spaced design when parameters are specified more or less correctly, the equally spaced design can be more robust regarding major parameter misspecifications.

### Online script for optimal designs

We created a Web application (based on the software R/shiny) which allows to obtain the optimal design for any of the three functions discussed here and for any values of EC50 and hill slope *b* and also provides the D-efficiency of any given design compared with the optimal design. The Web application is available under biostatistics.dkfz.de/DoseResponseDesigns/.

## Bayes optimal designs

As described in “Performance with misspecified parameters” section, the designs derived in “Log-logistic function” section to “Weibull function” section are only optimal for a specific set of parameters, which is unknown in practice. While a rough knowledge of the parameters usually is enough for a reasonably good design (see Table 2), larger uncertainty regarding the parameters will invalidate the approach. One possible solution is the Bayes optimal design approach (sometimes called quasi-Bayes, because it does not actually require a Bayesian data analysis). For a review of Bayes optimal designs, see, e.g., Chaloner and Verdinelli (1995). The idea here is that instead of specifying a single parameter guess to base the design on, a probability distribution of parameters is given instead (the a priori probability distribution).

As a simple example, the dose–response model parameter *b* might not be known exactly, but prior information might indicate that it would be found most likely at the values of 0.5, 1 or 2. Consequently, the design should do reasonably well in any of these three situations. If all three were considered equally likely, i.e., the a priori probability of each of the values is 33.3 %, the Bayes optimal design would be the one which maximizes the average information over these three scenarios.

Formally, this approach changes Eq. (1) in the equivalence theorem to

$$E\left[ {{\text{tr}}(A_{j} M^{ - 1} )} \right]/4 \le 1,\quad {\text{for}}\;{\text{all}}\;{\text{available}}\;{\text{dose}}\;{\text{levels}}\;j\;{\text{from}}\;1\;{\text{to}}\;m,$$

with *E*[] being the expected value of the term in brackets over all possible parameter values in the a priori distribution (see Atkinson and Cook 1995). In the example, $E\left[ {{\text{tr}}(A_{j} M^{ - 1} )} \right]$ would be the average of ${\text{tr}}(A_{j} M^{ - 1} )$ calculated for the three values of 0.5, 1 or 2 for *b*.

Similarly, Eq. (2) of the algorithm changes to

7 $$w_{j}^{i + 1} = w_{j}^{i} * E\left[ {{\text{tr}}(A_{j} M^{ - 1} )} \right]/4,\quad {\text{for}}\;{\text{all}}\;j\;{\text{from}}\;1\;{\text{to}}\;m.$$

Hence, the algorithm can be applied in exactly the same way as before using updating rule (7) instead of (5) for the weights. Note that only one set of dose levels can be used for the algorithm.

We will demonstrate the Bayes approach for the example discussed above, that is

1. the log-logistic model
2. an a priori probability for *b* of 1/3 each for values of 0.5, 1 and 2
3. all other parameters as in the standard parameterization
4. the set of dose levels corresponding to the standard parameterization *b* = 1.

**Fig. 3:**
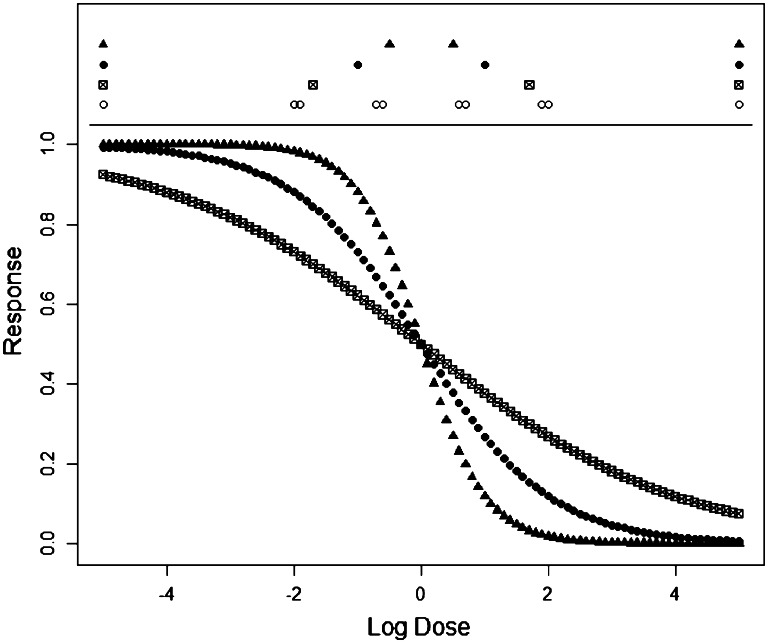
Log-logistic function for parameter *b* = 0.5 (*squares*), *b* = 1 (*solid dots*) and *b* = 2 (*triangles*), standard parameterization otherwise. *Top part* shows the dose levels included in the optimal design for these three parameter values using the same symbols, as well as for the Bayes optimal design shown as *circles*. Note that the weights are equal at 25 % for the fixed parameter designs, but varying for the Bayes design

Results for the other dose–response functions are structurally similar and not shown here. The different shapes of the log-logistic function under the three selected values of *b* are shown in the lower part of Fig. 3. Note that every symbol in the function plot corresponds to one of the 101 standard dose levels.

Running the algorithm with these settings results in the design shown in Table 3.

**Table 3:** Dose levels for a Bayes optimal design for three possible values of the parameter *b* in the log-logistic model

Log-logistic relationship										
	Log dose 1	Log dose 2	Log dose 3	Log dose 4	Log dose 5	Log dose 6	Log dose 7	Log dose 8	Log dose 9	Log dose 10
Dose	−5.0	−2.0	−1.9	−0.7	−0.6	0.6	0.7	1.9	2.0	5.0
Weight (%)	22.9	2.4	5.7	5.6	13.3	13.3	5.6	5.7	2.4	22.9

A priori distribution is *b* = 0.5 with 33.3 % probability, *b* = 1 with 33.3 % probability and *b* = 2 with 33.3 % probability

While the design retains the symmetry of the original design, many more dose levels are necessary and the relative weights are far less intuitive. Collapsing the four center pairs of dose levels to just four dose levels is possible but leads to a minor loss of efficiency of just 1.1 %. Further simplifying the design to a six-point design with dose levels of −5.0, −1.9, −0.6, 0.6, 1.9 and 5.0 with weights 20, 10, 20, 20 10 and 20 % does not result in a further loss of efficiency.

The top of Fig. 3 shows the single parameter variants of the designs on the standard dose level set for *b* = 0.5 as squares, *b* = 1 as solid dots and *b* = 2 as circles. As these are based on the standard dose levels (for *b* = 1), the designs are slightly different from the designs calculated on the transformed set of dose levels as described in “Log-logistic function” section [using (6) for transformation]. The dose levels indicated as circles show the Bayes optimal dose levels. The number of inner dose levels in the Bayes optimal design is increased, and the dose levels are more spread out than in the variants with fixed *b*. Furthermore, they are not simply combinations of the dose levels used in the single parameter designs. Thus, no generally applicable Bayes optimal designs can be proposed. Still, using the above approach, a suitable Bayes optimal design can be constructed for any specific practical situation.

In practice, the a priori distribution for *b* can also be a continuous distribution and does not have to be restricted to a small number of alternatives. In this situation, a Bayes optimal design can still be derived as described above.

## Comparison to the 3T3/NHK cytotoxicity testing guideline

The guidelines contained in the Background Review Document published by ICCVAM (Anon 2006) contain suggestions on dose level selections for cytotoxicity experiments. In Section 2.3.2.2 and Figs. 2 and 3 of this document, it is suggested that eight dose levels should be used at a weight of 10 % each and the vehicle control at weight 20 %. The dose closest to the calculated IC50 value in the range finder test should serve as the midpoint of the eight doses tested in the definitive test. The other dose levels should be equally distributed on the log scale in both directions. In the absence of other information (i.e., knowledge of the slope of the toxic response), the recommended dilution factor is 1.47. On a log dose scale, using a dilution factor of 1.47 corresponds to steps of log(1.47) = 0.38 each. Note that the guideline recommendation is similar to the equally spaced design considered before, but restricted to eight dose levels, a fixed spread factor and a fixed proportion of vehicle control measurements.

This basic recommendation is of course not useful in every situation, as the performance of a design greatly depends on the slope parameter *b*. For illustration, in our situation (standard parameterization, *b* = 1), the spread factor of 1.47 results in eight log doses only between about −1.5 and 1.5 in addition to the vehicle control, which is a highly concentrated design with an efficiency of only 48.4 % for the log-logistic model (log-normal model: 64.6 %; Weibull model: 80.5 %; required observations compared with 100 for optimum: log-logistic: 207; log-normal: 155; and Weibull: 125).

In the following, we investigate the potential performance of the guideline recommended approach of such a log equally spaced design. For this aim, we determine the spread factor which maximizes the efficiency while keeping the (log) dose levels within the range of −5.0 to 5.0. Under standard parameterization and in the log-logistic model, the optimal spread factor has a value of 3.0 and results in an efficiency of 76.2 % (132 observations). This result is intuitively plausible because a spread factor of 3.0 leads to dose levels less concentrated around the EC50. For the log-normal function, the optimal spread is 2.0 with an efficiency of 76.3 % (132 observations), and for the Weibull function, it is 1.7 with an efficiency of 86.1 % (117 observations).

For general *b*, the optimal spread factor for the guideline design can be calculated from (6) to be the standard spread factor to the power of 1/*b* for all dose–response functions considered here, i.e., 3.0^1/*b*^ for the log-logistic model, 2.0^1/*b*^ for the log-normal function and 1.7^1/*b*^ for the Weibull function. Consequently, the spread factor of 1.47 recommended in the guideline corresponds to a value for the hill slope of *b* = 2.85 for the log-logistic model. For the log-normal and Weibull functions, the corresponding *b*-values are 1.80 and 1.38, respectively.

Although one might expect that the guideline-based equally spaced design is more robust in the presence of parameter misspecifications, the comparison between the guideline-based design (with spread factor chosen based on hill slope) and the optimal design shows that the optimal design retains its superiority in all constellations considered in Table 2. Exemplarily, Table 4 compares the required number of observations of the optimal design for the log-logistic model (cf. Table 2) to number of observations of the guideline-based design in the same situations. The guideline-based designs stay suboptimal in all cases.

**Table 4:** Required sample sizes of the best 3T3 guideline-based design (with spread factor chosen based on hill slope) for the standard parameterization of the log-logistic function when the true parameters are not standard, compared with the optimal design with 100 observations in the same situation

True *b*	True *e*	Required *n* equally spaced design	Required *n* optimal design
1	1	131	100 (reference)
0.9	1	130	101
1	0.9	131	100
0.9	0.9	129	101
1.1	1.1	134	100
0.5	1	131	110
1	0.5	131	109
0.5	0.5	128	112
1	2	139	109
2	1	195	132
2	2	211	164

Parameters *c* and *d* do not affect the design

## Discussion

In this paper, we applied optimal design theory toward common problems regarding estimation of parameters in toxicological dose–response studies. Optimal designs were derived for three common dose–response functions, and a Web application was created which also provides the D-efficiency of any given design compared with the optimal design (available under biostatistics.dkfz.de/DoseResponseDesigns/).

In general, the resulting designs were similar for the three functions studied and suggested using control and only three dose levels, each on one quarter of the available replicates. Compared with the guideline recommended design for 3T3/NHK cytotoxicity testing, precision was increased by about 20 % in all cases.

Optimal designs require rough a priori estimates of the EC50 and the hill slope parameter, which can be obtained from a dose range finding study. Even if the a priori guesses of the parameters were wrong by a small or moderate amount, efficiency of the designs remained reasonably well. For large misspecifications, the optimal design loses efficiency. However, the same is true for the guideline design.

As the optimal designs are directly applicable to many practical situations, e.g., assays performed on 96-well plates, there are few reasons not to consider their use in practice. Even if the experimenter does not want to derive a formal design, the general structure (measurements at smallest and largest available dose plus the start and end of the approximately linear descent in the center) can serve as a useful rule of thumb for practical experiments.

In case that model parameters are associated with uncertainty, a Bayesian approach can be used. Under assumptions concerning probable values of the parameters, a new design can be derived. In our example, it became apparent that the design was similar in structure to the fixed parameter design, but the number of dose levels was increased and covered a slightly broader dose range.

Variants and extensions of optimal design theory exist for specific situations in toxicology. For example, to reconcile statistical optimal designs with practicality, Parker and Gennings (2008) suggest penalizing locally optimal designs with a desirability function. The desirability function is used to account for experimental features wished for by experimentalists. If such a desirability function was available, the approach by Gennings and Parker could be easily incorporated into the standard design algorithms used above.

Slob et al. (2005) proposes to favor designs with more than four dose groups to avoid the risk of unfavorable dose placements because of incorrect prior guesses of model parameters (see also Rhomberg 2005). They base their recommendation on simulation studies in which they evaluate the precision of an effect estimate under various dose response models. We investigated this situation using a Bayesian design approach and obtained more than four dose levels as well. In our example, using three potential values for *b*, the optimal Bayes design required six dose levels including control. With even more parameter uncertainty, even more dose levels might be needed. Thus, we can support the recommendation given by Slob et al. (2005), if major uncertainty regarding prior estimates is present.

In the present paper, we derived D-optimal designs that optimize precision of all parameter estimates. As discussed, for example, by Kuljus et al. (2006), the focus in toxicological studies may lie in optimizing the precision of a single parameter estimate, e.g., the EC50, or of a function of model parameters. Designs with this feature are called c-optimal designs. However, these designs often result in very few dose levels, which limit their practical use (see Pronzato 2009). Thus, further research in this area is needed.
